# Early validity and reliability evidence for the American Board of Emergency Medicine Virtual Oral Examination

**DOI:** 10.1002/aet2.10850

**Published:** 2023-03-26

**Authors:** Carl R. Chudnofsky, Earl J. Reisdorff, Kevin B. Joldersma, Kathleen C. Ruff, Deepi G. Goyal, Diane L. Gorgas

**Affiliations:** ^1^ Keck School of Medicine of the University of Southern California Los Angeles California USA; ^2^ American Board of Emergency Medicine East Lansing Michigan USA; ^3^ Mayo Clinic Rochester Minnesota USA; ^4^ Wexner Medical Center at The Ohio State University Columbus Ohio USA

## Abstract

**Background:**

The American Board of Emergency Medicine (ABEM) in‐person Oral Certification Examination (OCE) was halted abruptly in 2020 due to the COVID‐19 pandemic. The OCE was reconfigured to be administered in a virtual environment starting in December 2020.

**Objectives:**

The purpose of this investigation was to determine whether there was sufficient validity and reliability evidence to support the continued use of the ABEM virtual Oral Examination (VOE) for certification decisions.

**Methods:**

This retrospective, descriptive study used multiple data sources to provide validity evidence and reliability data. Validity evidence focused on test content, response processes, internal structure (e.g., internal consistency and item response theory), and the consequences of testing. A multifaceted Rasch reliability coefficient was used to measure reliability. Study data were from two 2019 in‐person OCEs and the first four VOE administrations.

**Results:**

There were 2279 physicians who took the 2019 in‐person OCE examination and 2153 physicians who took the VOE during the study period. Among the OCE group, 92.0% agreed or strongly agreed that the cases on the examination were cases that an emergency physician should be expected to see; 91.1% of the VOE group agreed or strongly agreed. A similar pattern of responses given to a question about whether the cases on the examination were cases that they had seen. Additional evidence of validity was obtained by the use of the EM Model, the process for case development, the use of think‐aloud protocols, and similar test performance patterns (e.g., pass rates). For reliability, the Rasch reliability coefficients for the OCE and the VOE during the study period were all >0.90.

**Conclusions:**

There was substantial validity evidence and reliability to support ongoing use of the ABEM VOE to make confident and defensible certification decisions.

## INTRODUCTION

Since its first administration in 1980, the American Board of Emergency Medicine (ABEM) Oral Certification Examination (OCE) had been delivered in an in‐person format. The ABEM OCE was typically conducted once in the spring and once in the fall each year. In‐person OCE administrations were halted abruptly in 2020 due to the COVID‐19 pandemic; the 2020 spring OCE and the 2020 fall OCE administrations were canceled. It soon became apparent that due to public health limitations and institutional travel bans, the resumption of an in‐person OCE was untenable. Consequently, ABEM reconfigured the OCE to be administered in a virtual environment. The first Virtual Oral Examination (VOE) was administered in December 2020.

Reliability is a necessary complement to validity. For the VOE, a sufficient level of reliability is required to demonstrate that the assessment is a credible measure that reflects a consistent level of quality. Reliability includes the ability of an assessment to have both reproducible results and high internal consistency. High‐reliability assessments have lower incidences of errors occurring by chance. Ideally, a high‐stakes examination such as a medical certification exam should have minimal error caused by extraneous factors (e.g., problems with consistent administration). For the past decade, the in‐person OCE was highly reliable (Cronbach *α* > 0.85) (ABEM, unpublished data, 2022).

Validity is an argument built on the evidence and experience resulting from the repeated use of an assessment. A validity argument supports the interpretation of an examination's results, as well as how those results are used, such as when making a decision about a physician's certification status. An assessment is valid if it measures what it is intended to measure and the assessment's scores can be interpreted as intended.^1^ For the purposes of this study, the authors modeled the types of validity evidence from the *Standards for Educational and Psychological Measurement* ^2^ that included test content, response processes, internal structure, and consequences of testing.^3^ The *Standards for Educational and Psychological Measurement* is the most widely used psychometric reference for assessing performance using high‐stakes testing.

As a certifying organization, ABEM has an interest in being confident that the VOE measures competencies that a board‐certified physician must have. Those who use the results of ABEM's assessments include certified physicians, hospital credentialers, state medical licensing boards, and the public who have a vested interest in certification being an accurate measure of a physician's capacity to deliver safe, high‐quality care. Like the in‐person OCE, the VOE must demonstrate that a physician has the competencies that contribute to providing such care.

Given the high‐stakes nature of specialty board certification, it was important to determine the degree to which the VOE was reliable as well as determine the presence of validity evidence to support the new format. The in‐person OCE had substantial validity evidence and reliability data to support its use in the certification of emergency physicians.^4^, ^5^, ^6^, ^7^, ^8^ The purpose of this investigation was to determine whether there is sufficient early validity and reliability evidence to support the continued use of the ABEM VOE for certification decisions.

## METHODS

This retrospective, descriptive study used multiple analyses to provide validity evidence and reliability support. Various data were used to gather different types of validity evidence for the VOE, including convergent data that compared the OCE and VOE. For reliability, multiple unique cohorts underwent the application of a multifaceted Rasch measurement. This study was deemed exempt from IRB review (Exemption #1) by The Ohio State University Office of Responsible Research Practices.

Data for this study were taken from the spring and fall 2019 in‐person OCEs, as well as the December 2020, March 2021, April 2021, and June 2021 VOEs. The OCE consisted of seven case administrations—five single‐patient cases and two multiple‐patient cases. Multiple‐patient cases included three patient encounters for each case. All “traditional” cases were scored in eight performance ratings using a 1–8 scale^9^ (Table 1). Traditional cases refer to typical case encounter format that ABEM uses. All traditional cases involved role playing, with the examiner assuming any necessary role (e.g., patient, parent, nurse, or consultant).

**TABLE 1:** Scoring parameters.

Traditional case^a^	Structured interview case^b^
Data acquisition	History
Problem solving	Physical
Patient management	Differential diagnosis
Resource utilization	Diagnostic testing
Health care provided (outcome)	Treatment
Interpersonal relations and communication skills	Final diagnosis
Comprehension of pathophysiology	Disposition
Clinical competence (overall)	Transitions of care

^a^ On the OCE, there were five single traditional cases and two triple cases with every case using the eight scoring parameters.

^b^ On the VOE, there were six single traditional cases scored with every case using the eight scoring parameters. There was also one structured interview case using approximately 25 scored points in the eight scoring domains.

The ABEM VOE consisted of seven encounters—six single‐patient cases and one structured interview (SI) case. There were no multiple‐patient cases. The SI case was a new type of case consisting of a conversation between the physician candidate and the ABEM examiner that was designed to ascertain a physician's thought processes. Throughout the interview case, test takers are given updated, determined, clinical, and diagnostic information to provide a uniform test administration experience. Pertinent clinical findings and diagnostic results are delivered through a variety of verbal, text, and photo stimuli. For the SI, there were 25 dichotomous points (0 or 1) distributed across eight scoring areas, for example, differential diagnosis and transition of care (Table 1).^10^ The SI scoring areas are modeled after physician tasks that routinely occur with nearly every patient encounter (e.g., taking a medical history, developing a differential diagnosis). The dichotomous scoring rubric and standardized script of administering the cases minimize subjective scoring and variability in case administration. The scoring approach is like that used by other medical specialty boards (i.e., American Board of Ophthalmology).^11^

The VOE required a sophisticated delivery platform that used Zoom Video Communications. An independent media vendor (Markey's) adapted Zoom to create a testing venue and testing schedule in a virtual environment that was similar to the prior in‐person examinations. Both test takers and examiners received “tech checks” to ensure that Internet devices and connections would function sufficiently during the VOE administration. Administration reliability was determined by the total number of cases that were dropped (e.g., due to technical interruptions or errors during the case administration).

### Content validity

Evidence for validity focused on multiple sources that included test content, response processes, internal structure, and consequences of testing.^3^ Evidence of test content validity was derived from a qualitative comparison of how the Model of the Clinical Practice of Emergency Medicine (EM Model) was applied to the OCE and to the VOE and the application of the ABEM examination blueprint^12^ and expert review of content and cognitive processes. Additional test content validity evidence was obtained by assessing physician skills required to clinically function in the emergency department and determining content relevancy through the survey. Given the limited number of cases seen by any individual candidate, ABEM relies on a case selection process that avoids an emphasis on any single focus of the EM Model. More importantly, the content area of assessment is less about covering a representative sample of disease conditions, but instead about assessing physician cognitive skills that are not substantially measured on the written, multiple‐choice question Qualifying Examination (e.g., data acquisition and resource utilization). All cases are extensively reviewed by a panel of experienced examiners and examination editors during initial development, prior to examination, and following each examination to ensure clinical relevance and content accuracy. This review included mock administrations of every case that could result in improvements to the case. Finally, although the content emphasis is not on disease conditions per se, it is important the content involving medical and traumatic conditions is relevant to clinical practice. When combined with the written, 305‐question Qualifying Examination, the VOE creates an assessment system that covers a substantial span of the EM Model based on a weighted content blueprint.^12^ Adjustments to the content blueprint have used several data sources including responses to detailed surveys (“job analyses”) by emergency physicians.

VOE cases (both “traditional cases” and structured interview cases) were created anew as well as modified previously administered cases. Modified content involved reformatting and updating cases from the eOral format (quasi‐simulation format) as well as case topics used in the paper‐only format that were used prior to the introduction of the eOral format in 2015.

The two post‐examination survey items of interest that assessed content relevance applied a 5‐point Likert scale (strongly disagree, disagree, neutral, agree, and strongly agree). The items were: (1) “Overall, the types of cases on this examination were cases that an emergency physician should be expected to see”; and (2) “In my practice, I have seen most of these cases.” These survey items were developed by an expert panel of clinically active emergency medicine experts and have been used to support validity claims on prior OCEs.^5^

### Response process

Validity evidence for response processes included a clear chain‐of‐reasoning of assessed skills that are germane to providing emergency medical care. Think‐aloud protocols are used in the SI case whereby physicians are asked to explain their rationales for certain responses and decisions. Finally, expert‐novice studies were indirectly obtained by analyzing point measure correlations that demonstrated that better test takers consistently performed better on the cases.

### Internal structure

Evidence of validity through internal structure was determined by the degree of internal consistency as measured by Rasch reliability coefficients. Item response theory (IRT) was supported by the way case difficulty was maintained over time. This property allowed the use of IRT to equate examinations. Equating the VOE allowed for the ability to compensate for VOEs that differed in the degree of difficulty between examinations.

Additional internal structure validity evidence involved test takers' performance patterns (e.g., pass rates and distribution tendencies) for the VOE compared with the OCE. Specifically, performance distributions (score distributions) for the OCE were compared with the VOE distributions.

### Consequences of testing

Evidence for the consequences of testing were determined by pass rates as well as the marketability of certified physicians and the value that hospital systems and credentials placed on the resulting certificate.

Another dimension of consequential validity was determined based on how the examination results are used, not the examination itself. The ability to use a test's results to make a high‐stakes certification decision supports consequential validity. Further consequential validity support of the assessment comes from third‐party use of the credential that is determined by the assessment. Specifically, the way academic departments, hospital systems, physician employers (including the military), and the public would view certification obtained through the VOE could also support consequential validity arguments.

### Reliability

To determine the reliability of the VOE, a multifaceted Rasch measurement was used.^13^ This analytic tool is part of a family of mathematical models (item response theory) that attempt to explain the relationship between a latent trait (e.g., cognitive skill and medical knowledge) and performance on a test (e.g., the VOE). The Rasch model describes reliability as the observed variance of item (or case) difficulty measures and the mean of squared standard errors of item difficulty measures. Using this approach, reliability would be higher by increasing the heterogeneity of physician performance and having a larger cohort of test takers.

### Analysis

The data were largely descriptive (e.g., survey response frequencies), with Chi square testing to evaluate nominal values. Rasch reliability coefficients were calculated as a byproduct of fitting a multifaceted Rasch model to the examination data. ABEM conducted a post‐hoc analysis of the OCE using the Rasch method to compare reliability between the OCE and VOE using a similar methodology. A priori parameters for reliability were determined to be good (0.80–0.89) and excellent (0.90–0.99), which are typical psychometric thresholds for most measures of reliability.^14^ All data were deposited in a highly secure ABEM server and all data reports used aggregate, deidentified, and not re‐identifiable data.

## RESULTS

There were 2279 physicians who took the 2019 in‐person OCE examination and 2153 physicians who took the VOE during the study period (Table 2). Of these, 1791 physicians in the OCE group (78.6% response rate) addressed the statement, “Overall, the types of cases on this examination were cases that an emergency physician should be expected to see”; 1235 physicians in the VOE group (57.4% response rate) also addressed the statement (Table 3). Of the OCE group, 92.0% agreed or strongly agreed with the statement, compared with 91.1% of the VOE group. Though this combined agreement level was close between the OCE and VOE, the strength of endorsement of the statement was significantly different statistically. VOE test takers had a higher rate of “strongly agree” responses (42.1% vs. 26.3%).

**TABLE 2:** Physician participation.

Format	Examination	Test takers, *N*	Evaluations, *N*	Response rate (%)
In‐person oral certifying exam	Spring 2019	1151	959	83.3
	Fall 2019	1128	846	75.0
Subtotal	–	2279	1805	79.2
Virtual oral examination	December 2020	196	125	63.8
	March 2021	504	281	55.8
	April 2021	781	446	57.1
	June 2021	672	386	57.4
Subtotal	–	2153	1238	57.5
Total	–	4432	3043	68.7

**TABLE 3:** Comparison of virtual versus in‐person agreement to expected case relevancy.

	Strongly disagree *n* (%)	Disagree *n* (%)	Neutral *n* (%)	Agree *n* (%)	Strongly agree *n* (%)
Virtual total (*n* = 1235)	15 (1.2)	14 (1.1)	81 (6.6)	593 (48.0)	532 (43.1)
In‐person total (*n* = 1791)	9 (0.5)	53 (3.0)	82 (4.6)	1176 (65.7)	471 (26.3)

*Note*: Cases expected to see: “Overall, the types of cases on this examination were cases that an emergency physicians should be expected to see.” 2 × 5 chi = square test; *p* < 0.001.

There were 1796 physicians in the OCE group (78.8% response rate) and 1238 (57.5% response rate) in the VOE group who addressed the statement, “In my practice, I have seen most of these cases” (Table 4). The OCE group was more likely to agree or strongly agree with the statement than the VOE group (89.1% vs. 85.4%; *p* < 0.001). VOE test takers had a higher rate of strongly agree responses (32.6% vs. 21.0%; *p* < 0.001).

**TABLE 4:** Comparison of virtual versus in‐person agreement to in‐practice case relevancy.

	Strongly disagree *n* (%)	Disagree *n* (%)	Neutral *n* (%)	Agree *n* (%)	Strongly agree *n* (%)
Virtual total (*n* = 1238)	16 (1.3)	48 (3.9)	117 (9.5)	654 (52.8)	403 (32.6)
In‐person total (*n* = 1796)	12 (0.7)	76 (4.2)	108 (6.0)	1206 (67.1)	394 (21.9)

*Note*: Cases actually seen: “In my practice, I have seen most of these cases.” 2 × 5 chi = square test; *p* < 0.001.

Evidence supporting test content validity of the VOE was found in the continued use of the EM Model as the source for all VOE material.^15^ The EM Model was developed through a multi‐organizational consensus process. Every 3 years, most major emergency medicine organizations convene a task force to review and revise the EM Model. The EM Model is posted publicly and recommendations for changes are solicited from all major emergency medicine organizations and from ABEM‐certified physicians. In addition, many of the cognitive processes that are assessed are related to the list of physician tasks contained in the EM Model. The process of case development for the VOE involved the same processes as for the OCE, which involved multiple iterations of expert content review and focused on assessing the cognitive processes that a physician would use when caring for a patient in the emergency department.

Response process focused on assessing the reasoning that a physician used in working through a case. Skills such as obtaining a medical history and gathering data from a physical examination are assessed, which are axiomatic when caring for a patient with undifferentiated complaints. Diagnostic uncertainty is often clarified using a think‐aloud protocol whereby the physician is asked to role play or state a specific diagnosis or provide the interpretation of a diagnostic study (e.g., describing a radiographic finding).

Validity support for the VOE was also evident in the new type of case that was used—the SI. This case format was a conversation between the examiner and test taker in which test takers were regularly asked for the rationale for their responses to specific questions. This interrogatory approach can better assess a physician's thought processes and logic, which is a think‐aloud protocol.

Of note, several other medical certifying boards have used this approach successfully to determine certification status (e.g., American Board of Ophthalmology). While the content of each specialty is substantially different, the fact that multiple national medical specialty boards find this format suitable to make certification decisions lends a degree of credibility to the format.

For reliability, the Rasch coefficients for the OCE and the VOE during the study period were all >0.90 (Table 5). The range of mean scores on the OCE administrations was 5.52 to 5.59, compared with 5.56–5.69 for the VOE. Other distributional features that were similar included the following: SDs (OCE: 0.25–0.26, VOE: 0.25–0.27), skew (OCE: −0.32 to −0.06, VOE: −0.40 to +0.09), and kurtosis (OCE: 3.10–3.25, VOE: 3.07–3.48). Having similar reliability statistics, distributional characteristics, and pass rates demonstrates evidence of internal structure validity.

**TABLE 5:** Rasch reliability coefficients for each administration.

Examination	Rasch reliability coefficient
Spring 2019	0.95
Fall 2019	0.93
December 2020	0.92
March 2021	0.94
April 2021	0.98
June 2021	0.96

The pass rate for the OCE ranged from 93% to 94% (average 94%) with a 20‐year pass rate range of 88% to 98%. The pass rate for the VOE administrations ranged from 90% to 95% (average 92%). ABEM used the same convention for establishing a passing score for the VOE as it has always used. ABEM assembled a diverse panel of clinically active emergency physicians to undergo a modified Angoff standard‐setting process. This approach to standard setting is widely used by medical certifying boards. There were no difficulties in establishing a proposed passing score for the VOE relative to prior OCE administrations. For administration reliability, there were 53 VOE cases dropped from scoring due to administration errors, out of 15,022 cases delivered, for a 0.35% case drop rate (1 case dropped for every 283 cases administered). The OCE case drop rate was approximately 0.15%.

ABEM used both new and experienced oral examiners to administer cases. New examiners did not administer structured interview cases. The number of new examiners was similar to the number used in prior examinations. Though not specifically measured, examiners did not express any significant difficulty administering the cases. The ability to administer and score the cases using the long‐established scoring parameters for the traditional cases as well as the new approach using the structured interview format was easily accomplished as evidenced by the completion of the scoring rubrics. All examiners, regardless of experience, undergo direct observation to ensure that the examination is being administered according to ABEM standards. Among examiners, there is sometimes diversity of hawkishness in scoring. ABEM maintains scoring fairness by adjusting scoring through the Rasch model of IRT.

## DISCUSSION

This study is the first report of validity and reliability evidence for the virtual format of the ABEM certifying examination. Prior studies of the in‐person OCE showed substantial validity and reliability support.^4^, ^5^, ^6^, ^7^, ^8^ To maintain a similarly high level of validity and reliability, ABEM designed the VOE to be similar to the OCE in content and administration. The OCE had amassed substantial validity evidence as previously reported. Specifically, content validity support came from the use of the EM Model and the case development process as well as the psychometric performance of the examination cases.^4^, ^5^, ^6^, ^7^, ^8^ In addition, the OCE amassed consequential validity by the use of ABEM certification as a criterion for hiring or a promotion in community and academic practice settings.

Establishing validity is an iterative process whereby evidence is accumulated over time and through multiple experiences, including interpretations of assessments and decisions that are based on assessment results. Within the field of psychometrics, there were two dominant frameworks for determining the validity of an assessment: Messick's model and Kane's model.^16^, ^17^ A more contemporary approach to validity focuses on the evidence for validity found in test content, response processes, internal structure, relations to other variables, and consequences of testing. For this investigation, not all forms of validity evidence were sought; specifically, evidence obtained by relations to other variables was not gathered. To determine whether the VOE succeeded in demonstrating early validity evidence, this study sought to provide evidence that aligned with this contemporary framework. Certain elements of validity evidence will be stronger than others and some types of validity evidence require an assessment of clinical performance, which can take years to obtain.

Test content validity evidence was supported by the use of the EM Model in developing VOE content. All VOE content was contained in the EM Model. The EM Model is publicly available and used to define educational content, including residency curricula.^18^, ^19^, ^20^ Basing the VOE in the EM Model provides substantial content validity evidence. In addition, substantial evidence of test content was provided through the way the EM Model was developed initially and is amended regularly—the EM Model's stability of form and content over time, its alignment with detailed specialty‐specific surveys, and its use for multiple similar assessments in emergency medicine.^21^ Validity evidence was also provided by the VOE's psychometric performance, such as pass rates and distribution indices, compared with the OCE.

Evidence for test content validity was provided by the physician survey responses regarding the types of clinical cases that an emergency physician has seen or is expected to see. Responses were measured for the VOE, as well as compared with prior OCE survey responses. More than 90% of physicians confirmed the relevance of VOE cases as akin to cases seen in clinical practice, which provided additional evidence of “face validity” and content validity for the VOE. Of note, the frequency of agreement responses regarding the case that a physician has seen was high, which supports assertions of content relevance. This finding provides additional test content validity evidence. Though survey results were relatively close in frequency, the OCE had a statistically significant higher rate of agreement. The importance of this statistical result is uncertain as a practical finding.

Validity evidence based on the internal structure of the VOE was obtained largely through measuring reliability. The Rasch reliability coefficients demonstrate excellent internal consistency. The ability to equate each administration via the application of item response theory further supports the validity of the internal structure of the VOE. Moreover, equating allowed the maintenance of an inter‐examination difficulty scale, which provided additional validity evidence based on internal structure.

When apply evidence for the consequences of testing (consequential validity), the VOE scores were used to determine ABEM certification status. ABEM certification achieved by passing the VOE was used by third parties to make hiring and promotion decisions. These certification decisions made based on candidate performance on the VOE were identical to decisions made on the basis of candidate performance on the OCE even though the test formats varied. The in‐person OCE has had decades of use in the marketplace with consistent and generalizable results from one cohort to another supports consequential validity. By hiring physicians who were certified through the VOE process, the market confirmed that certification awarded by passing the VOE was a sufficient credential. There has been insufficient time to study the hiring of physicians who have been certified through the VOE process compared to the OCE process. ABEM is unaware of any instances where the certification credential obtained through the VOE has been, by itself, insufficient for employment or hospital credentialing. The similarity in psychometric performance between the VOE and OCE, as well as other forms of validity evidence, supported ABEM's decision to make confident, defensible determinations about certification status.

To address external validity, or the ability to generalize from one population of test takers to another, performance patterns were compared between the OCE and VOE. There was overlap of mean scores on the OCE and the VOE, which demonstrates a degree of test‐to‐test consistency in performance. Likewise, similar passing rates indicated a degree of consistency. In general, hospital systems and the public do not know the details of ABEM certification. Nonetheless, ABEM captures nearly all test performance information on physicians completing emergency medicine residencies including the distribution of scores on tests and OCE performance ratings. This multiyear, global performance knowledge is used when making a final determination on the passing standard (passing score) of the Oral Examination. Further supporting the psychometric rigor of ABEM's processes is that ABEM is the only medical specialty board within the American Board of Medical Specialties to receive accreditation by the National Commission for Certifying Agencies.

To provide ABEM with the defensible use of the OCE for certification decisions, which also supports the prior valid use of the OCE, ABEM used standard setting to determine the performance standard (i.e., passing score). The same process was again used before making certification decisions using the VOE. Standard setting involved an independent panel of clinically active emergency physicians who reviewed every scored element of every VOE case and proposed a passing score using an extension of the modified Angoff method.^22^ This panel consists of a stratified, randomly selected group of examiners who are representative of the specialty at large using criteria that includes practice type (academic and community) and demographic data (gender, race/ethnicity, and geographic location). The modified Angoff method is used to provide the ABEM Board of Directors with an empirically derived score on which to make certification decisions. Variations of the Angoff method are used widely to establish the passing standard (passing score) for a high‐stakes assessment. In support of using evidence for response processes and internal structure, there were no difficulties in applying ABEM's usual procedures to determine a passing scoring for the VOE. Also, by maintaining the use of the EM Model as the basis for content (including the Physician Task component), ABEM can be assured that its interpretation of the passing score for the VOE (i.e., the physician has the capacity to deliver safe, high‐quality care) is unchanged from the OCE.

The Rasch coefficients for the VOE provided evidence of excellent reliability. The Rasch method was used because the Rasch approach uses the actual average error of measurement variance of a latent trait measured by the VOE (e.g., cognitive skill and medical knowledge). Other measures of reliability use respondents' test scores in calculating the observed variance, which can be misleading because test scores are a nonlinear representation of the underlying latent trait's variability. Rasch tends to be a more conservative approach and thus minimizes the risk of overestimating internal consistency (reliability). An additional advantage of the multifaceted Rasch model is that the approach considers multiple parameters such as rater severity, task difficulty, and test‐taker ability. The Rasch reliability coefficients for the 2019 spring and fall OCEs were 0.95 and 0.93, respectively (ABEM, unpublished data, 2019), which were comparable to the VOE results.

The new SI case requires special mention. Many member boards of the American Board of Medical Specialties use the SI case to make certification decisions and have done so for decades. ABEM added the SI to augment the types of competencies that it could measure. For example, the traditional case format did not always allow the examiner to understand why a physician did certain things (e.g., ordered an imaging study). Understanding a physician's thoughts supporting an action provided new and different information that ABEM used to make certification decisions. The additional information was based on activities that are performed routinely in clinical practice, which also provided content and construct validity evidence for the SI. Moreover, having questions that address these commonly used skills expands the VOE's coverage of the EM Model, particularly around physician tasks. The addition of more EM Model content adds construct validity to the VOE. The high reliability associated with the VOE and SI is likely due to the rigorous training of oral examiners, as well as the standardized approach to scoring that includes clear definitions of correct and incorrect responses.

## LIMITATIONS

Establishing validity for the VOE is a long‐term and ongoing activity requiring the appropriate use of the assessment and resultant certification. Acquiring additional validity evidence will create greater confidence in physicians, physician employers, and the public about the use of the VOE for ABEM certification.

The data and validity evidence are early and limited. Additional experience may demonstrate differences not identified in this early study. As early career physicians become more familiar with the format of the SI and test performance changes, the validity and reliability evidence could change.

There was a large response rate difference between the OCE and VOE. It is possible that the VOE had a lower response rate because test takers received the survey by email after the examination, while at the in‐person examination, test takers received paper surveys that they would have to walk past to exit the examination venue. Despite this discrepancy, the sample sizes for both formats were large and probably overcomes concerns about sample size and self‐selection bias.

The survey questions about case relevancy were “agreement” questions that lend themselves to an affirmative response bias. Whether such bias was present is less important than any difference between the two exam formats. The same questions were used on both formats, allowing for an accurate comparison. Although the responses were statistically different to a significant degree, the general level of agreement was similar.

Another limitation to the analyses mentioned in this study is the lack of inter‐rater reliability statistics. ABEM specifically chose to forgo its typical observer rating program for the initial phase of the VOE. Every examiner did undergo direct observation by an examination leader to observe that the case was being administered to ABEM standards. Every scored result was reviewed to ensure adherence to case scoring guidelines.

Finally, because the VOE was implemented after a 1‐year delay, it is possible that there could have been an impact on the experience. Clearly, a different format mandated a different examiner and candidate experience. Despite any potential impact, the VOE was able to be administered at a record rate and led to results that were consistent with prior test administrations—any consequential impact was not apparent.

## CONCLUSIONS

There is substantial early validity evidence to support ongoing use of the ABEM VOE to make confident and defensible certification decisions. There are also strong reliability data to support confidence in the reproducibility and consistency fairness of the VOE format.

## AUTHOR CONTRIBUTIONS

Study concept and design: EJR, KBJ, CRC, DLG. Acquisition of the data: KBJ. Analysis and interpretation of the data: KBJ, EJR. Drafting of the manuscript: EJR. Critical revision of the manuscript for important intellectual content: CRC, EJR, KBJ, KCR, DLG. Statistical expertise: KBJ. Acquisition of funding:

## CONFLICT OF INTEREST STATEMENT

CRC is a former director of the American Board of Emergency Medicine. DGG, and DLG are directors of the American Board of Emergency Medicine Board of Directors. EJR, KBJ, and KCR are employed by the American Board of Emergency Medicine.
